# Swimming Motility in a Longitudinal Collection of Clinical Isolates of *Burkholderia cepacia* Complex Bacteria from People with Cystic Fibrosis

**DOI:** 10.1371/journal.pone.0106428

**Published:** 2014-09-09

**Authors:** James E. A. Zlosnik, Paul Y. Mori, Derek To, James Leung, Trevor J. Hird, David P. Speert

**Affiliations:** Centre for Understanding and Preventing Infection in Children, Department of Pediatrics, Faculty of Medicine, University of British Columbia, Vancouver, British Columbia, Canada; Ghent University, Belgium

## Abstract

Chronic bacterial lung infections in cystic fibrosis (CF) are the leading cause of morbidity and mortality. While a range of bacteria are known to be capable of establishing residence in the CF lung, only a small number have a clearly established link to deteriorating clinical status. The two bacteria with the clearest roles in CF lung disease are *Pseudomonas aeruginosa* and bacteria belonging to the *Burkholderia cepacia* complex (BCC). A number of common adaptations by *P. aeruginosa* strains to chronic lung infection in CF have been well described. Typically, initial isolates of *P. aeruginosa* are nonmucoid and display a range of putative virulence determinants. Upon establishment of chronic infection, subsequent isolates ultimately show a reduction in putative virulence determinants, including swimming motility, along with an acquisition of the mucoid phenotype and increased levels of antimicrobial resistance. Infections by BCC are marked by an unpredictable, but typically worse, clinical outcome. However, in contrast to *P. aeruginosa* infections in CF, studies describing adaptive changes in BCC bacterial phenotype during chronic lung infections are far more limited. To further enhance our understanding of chronic lung infections by BCC bacteria in CF, we assessed the swimming motility phenotype in 551 isolates of BCC bacteria from cystic fibrosis (CF) lung infections between 1981 and 2007. These data suggest that swimming motility is not typically lost by BCC during chronic infection, unlike as seen in *P. aeruginosa* infections. Furthermore, while we observed a statistically significant link between mucoidy and motility, we did not detect any link between motility phenotype and clinical outcome. These studies highlight the need for further work to understand the adaptive changes of BCC bacteria during chronic infection in the CF lung.

## Introduction

People with cystic fibrosis (CF) experience chronic lung infections, lasting years and sometimes decades. These lung infections are the main cause of morbidity and mortality in CF, leading to either lung transplantation or death through cumulative damage to the lungs. A number of bacterial species are associated with chronic infection in the CF endobronchial space [1], [2]. Bacteria belonging to the *Burkholderia cepacia* complex (BCC) are particularly concerning to the CF community due to their well-documented capacity for inter-patient transmission and an association with an unpredictable but typically worse outcome [1], [3], [4]. The BCC comprises at least 18 distinct species of bacteria and all, except for *B. ubonensis*, have been isolated from the lungs of people with CF [5], [6]. Historically, *B. cenocepacia* has been the most common and problematic species in many CF centres. However, the epidemiology of BCC bacteria in CF appears to be changing, with *B. multivorans* becoming the most common cause of new BCC infection in CF in number of studies [1], [7]

The most common pathogen in CF is *Pseudomonas aeruginosa* [1]. Numerous studies have described a range of common adaptations to chronic infection by *P. aeruginosa*. The current paradigm for *P. aeruginosa* infections in CF involves infection with a virulent nonmucoid strain, which, upon establishment of chronic infection, ultimately converts to the mucoid phenotype through the production of exopolysaccharide alginate [8]. In addition, a range of other adaptations have been described, which presumably enhance the capacity for survival in the CF lung [9], [10]. These often include the loss of a range of putative virulence determinants such as a quorum sensing and swimming motility as well as acquisition of other traits such as increased antibiotic resistance, serum sensitivity through loss of LPS O-polysaccharide and hypermutability [10]–[15].

Currently published data suggest that the pathogenic mechanisms employed by BCC bacteria in chronic CF lung infections may in fact be distinct from those observed in *P. aeruginosa*. For instance, a recent survey of sequential clinical isolates from a number of CF patients attending the Vancouver CF clinics who had been chronically infected with BCC indicated that, unlike *P. aeruginosa*, BCC maintain quorum sensing systems during chronic infection in the CF lung [16]. Recent transcriptomic and proteomic studies on isolates of *B. cenocepacia* from a single chronic infection have also suggested that virulence may be increased in isolates taken from later in chronic infection [17], [18]. In addition to these experimental data, the apparent aggressiveness of BCC bacteria in CF is also consistent with BCC bacteria deploying differential pathogenic mechanisms during chronic infections. Most notable is the well described capacity for BCC to cause a typically fatal invasive necrotizing pneumonia known as ‘cepacia syndrome’, which has commonly been associated with *B. cenocepacia* but is also known to occur in other species including *B. multivorans* and *B. dolosa* [19]–[21]. Invasive disease is rarely, if ever, seen in *P. aeruginosa* infections in CF.

In contrast to *P. aeruginosa*, studies on adaptation to chronic lung infections in CF by BCC are very limited. We have previously shown that, unlike *P. aeruginosa*, the nonmucoid phenotype is associated with a worse outcome in terms of lung function decline and survival, while mucoid to nonmucoid transitions are common in adaptation to chronic infection [22], [23]. Other studies have also documented BCC phenotypic colonial morphology variation in the context of chronic CF lung infections [24]. Variation in some other phenotypic traits has been described in clinical isolates of BCC bacteria, most notably, in antimicrobial susceptibility profiles, in the context of chronic CF infections [25], [26]. Recently, whole genome sequencing of sequential isolates from multiple patients in an outbreak of *B. dolosa* in the United States also revealed a number of candidate pathogenicity genes that appear to be commonly mutated during chronic infection and may confer elevated virulence on this species [27].

We have previously described an association between colonial morphology and motility, whereby increased flagellin protein production and swimming motility were detected in a shiny colony clonal variant that was obtained from an infection model where the infection had been established with an isolate that demonstrated a matte colony morphology [28], [29]. The swimming motility phenotype is also of interest, due to the well described capacity for bacterial flagella to stimulate inflammation through the TLR5 pathogen recognition receptor as well as its involvement in epithelial invasion by BCC bacteria [30]–[34]. In this study we examine the clinical significance of motility in BCC bacteria. We report the largest assessment to date of swimming motility from a longitudinal collection of over 550 BCC isolates, collected at least annually from 100 CF patients attending the Vancouver clinics during the period June 1981–June 2007.

## Materials and Methods

### Bacterial isolates

Bacteria were collected from CF patients at least annually between June 1981 and June 2007 from the Vancouver CF clinics as described previously [23]. Briefly we examined 551 isolates from 100 patients. For patients culturing BCC this collection typically contains at least one isolate per year. Bacteria were routinely stored by freezing confluent growth, taken from a purity plate, at −80°C in Mueller Hinton broth (Oxoid) plus 8% dimethyl sulfoxide. For experiments, bacteria were revived on a Columbia Blood Agar plate, and a fresh culture was established the day prior to the experiment on a Luria Bertani (LB) agar plate (10 g l^−1^ tryptone, 5 g l^−1^ yeast extract, 10 g l^−1^ sodium chloride) containing 1.5% agar. All plates were incubated overnight at 35°C.

### Swimming motility

Swimming motility for each isolate was individually assessed on LB supplemented with 0.3% agar plates as previously described [11]. Briefly, plates were poured on the bench with no drying time and then used the same day. Growth from the confluent area of a freshly cultured plate was inoculated into the center of the swimming motility agar plates using a sterilized wooden stick. Motility plates were then incubated for 48 hours at 35°C in stacks 4 plates high, the plates were kept upright and not inverted. At 48 hours the diameter of the colonial growth was measured. Isolates with less than or equal to 10 mm growth at 48 hours were classified as nonmotile, while those with >10 mm growth were classified as motile. Motile and nonmotile controls, *P. aeruginosa* PAK and *P. aeruginosa* PAK Δ*fliC* [35], were run in all experiments.

### Western blotting

Whole protein extractions from BCC bacteria were prepared as described previously [11]. Briefly, bacterial growth was scraped from the confluent area of a freshly grown agar plate using a sterile wooden stick and resuspended in 1 ml of 1.5 mM Tris.Cl pH 7.5. The bacteria were then added to an equal volume of 0.1 mm diameter glass beads in a 2 ml screw cap centrifuge tube (Lysing Matrix B, MP Biomedicals). Bacterial cells were then broken with 3 rounds of beating in a FastPrep ribolyzer (ThermoSavant) set to speed 6.5 for 45 seconds. Cellular debris was pelleted by centrifugation at 18,400 x *g* in a Hettich Mikro 200 microcentrifuge. 250 µl of supernatant was then removed to harvest the extracted protein. Protein concentration was determined using the Micro BCA protein assay kit (Pierce). 15 or 25 µg of protein was separated on a 12% acrylamide gel, proteins were transferred to a PVDF membrane by electroblotting and then probed using a 1 in 10,000 dilution of an anti-*Burkholderia pseudomallei* flagellin antibody [36] as described previously [37]. Blots were visualized either using chemiluminensce (Amersham ECL detection kit, GE Healthcare) with a 1 in 10,000 dilution of an anti-rabbit HRP linked secondary antibody (Cell Signalling) or green fluorescence using a 1 in 10,000 dilution of a goat anti-rabbit IRDye 800CW secondary antibody (Licor) on an Odyssey (Licor) infrared imaging system. Blots were performed in at least triplicate for all isolates examined.

### TLR5 stimulatory assays

TLR5 activation was assessed using a TLR5-NF-κB luciferase reporter system as described previously [32]. Briefly, bacterial cells were grown overnight in 3 mls of LB broth at 37°C on an orbital rotator. Bacterial cells were harvested by centrifugation (2,340 x *g* for 5 minutes) and washed twice in phosphate buffered saline and adjusted to 2.5×10^6^ CFU ml^−1^. Heat killed bacteria (30 mins at 65°C) were then added to 5×10^4^ freshly cultured CHO-K1 cells containing the TLR5-NF-κB luciferase reporter, representing an MOI of 50∶1, and incubated for 4 hrs in 96-well plates. CHO-K1 cells were routinely cultured both before and during experiments in F-12 K media (Gibco). Activation of the TLR5-NF-κB luciferase reporter was then assessed using a luciferase assay (Promega) and a Tecan Infinite M200 plate reader. Luminescence was measured by injecting 50 µl of substrate into each well, with an injection speed of 200 µl sec ^−1^, and luminescence was measured over an integration time of 5 seconds. All values are expressed relative to an unstimulated control containing only the reporter cell line and no bacteria. TLR5 activation assays were performed on five separate occasions for each isolate and in triplicate on each occasion.

### Clinical data

Clinical data were previously collected for all patients included in the study [22]. We employed the same inclusion/exclusion criteria in this study. The clinical data included in this manuscript was obtained following approval the Providence Healthcare Research Ethics Board - part of the UBC Research Ethics Boards (H07-01396). Informed consent was not required as the data was analysed in aggregate and de-linked from any patient identifiers.

### Statistical analysis

All motility data were collected and stored in a Microsoft Access 2010 database. For analysis, data were exported initially to Microsoft Excel 2010 and then transferred to GraphPad Prism V.4. Data were graphed using either Microsoft Excel 2010 directly or exported to GraphPad Prism V.4. All statistical analyses were performed with appropriate statistical tests using GraphPad Prism V.4.

## Results

### Prevalence of the motile phenotype in clinical isolates of BCC in the Vancouver CF population 1981–2007

**Figure 1:**
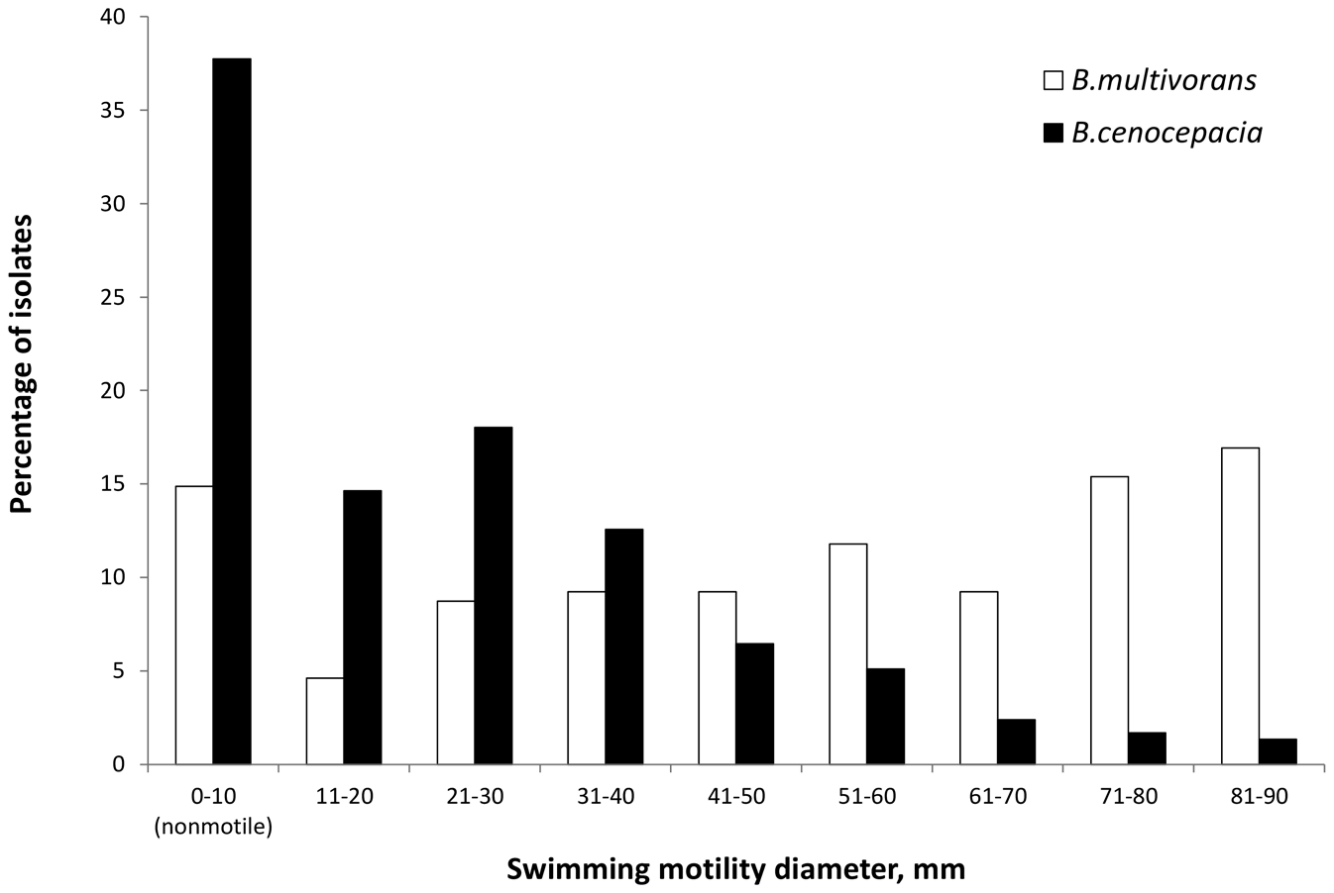
Analysis of swimming motility capacity of *B*. *multivorans* (white bars) and *B. cenocepacia* (black bars) isolates from cystic fibrosis lung infections in Vancouver between 1981 and 2008. Motility was assessed by inoculating bacteria into the center of a motility agar plate and incubating for 48 hrs, after which the diameter was measured. Isolates were classified as motile if colonial diameter was >10 mm and nonmotile if the diameter was less than or equal to 10 mm.

Isolates from people with CF infected with BCC are routinely collected and stored in our laboratory at least annually. We have previously reported data from this collection, describing the prevalence of the mucoid phenotype in Vancouver isolates of BCC from CF infections between June 1981 and June 2007 [23]. Given the well-described loss of swimming motility in *P. aeruginosa* during chronic CF lung infections, we assessed our isolate collection for swimming motility in order to understand the significance of swimming motility during chronic BCC infections in CF. An examination of the full isolate collection revealed that both motile and nonmotile isolates of all species were present, with the exception of *B. cepacia* for which there were only four isolates that were all motile and *B. metallica* for which the sole isolate was motile (Table 1). In total we examined 551 isolates from 100 patients. *B. cenocepacia* is traditionally considered the most virulent of the BCC species, so it is notable that a larger percentage - 38% - of *B. cenocepacia* isolates examined were nonmotile as opposed to 14% of *B. multivorans* (Table 1). We also assessed the extent of motility displayed by these isolates, in terms of the diameter of the resultant colony when *B. cenocepacia* and *B. multivorans* were grown on swimming motility agar. These data show that, consistent with this trend, *B. cenocepacia* isolates on average exhibit considerably smaller diameter of colonial growth than *B. multivorans* on swim plates (Fig 1). In fact only 1% of *B. cenocepacia* isolates in our collection possessed the capacity to swim across the entire plate, whereas 16% of *B. multivorans* isolates possessed this capacity (Fig 1).

**Table 1:** Prevalence of swimming motility in isolates of *B. cepacia complex* from cystic fibrosis infections in Vancouver from 1981–2008.

	Total number of isolates tested, *n*	Nonmotile, *n* (%)	Motile, *n* (%)
*B. cepacia*	4	0 (0)	4 (100)
*B. multivorans*	207	29 (14)	178 (86)
*B. cenocepacia*	294	111 (38)	183 (62)
*B. stabilis*	10	3 (30)	7 (70)
*B. vietnamiensis*	35	23 (66)	12 (34)
*B. metallica*	1	0 (0)	1 (100)

### Motility and the mucoid phenotype

**Figure 2:**
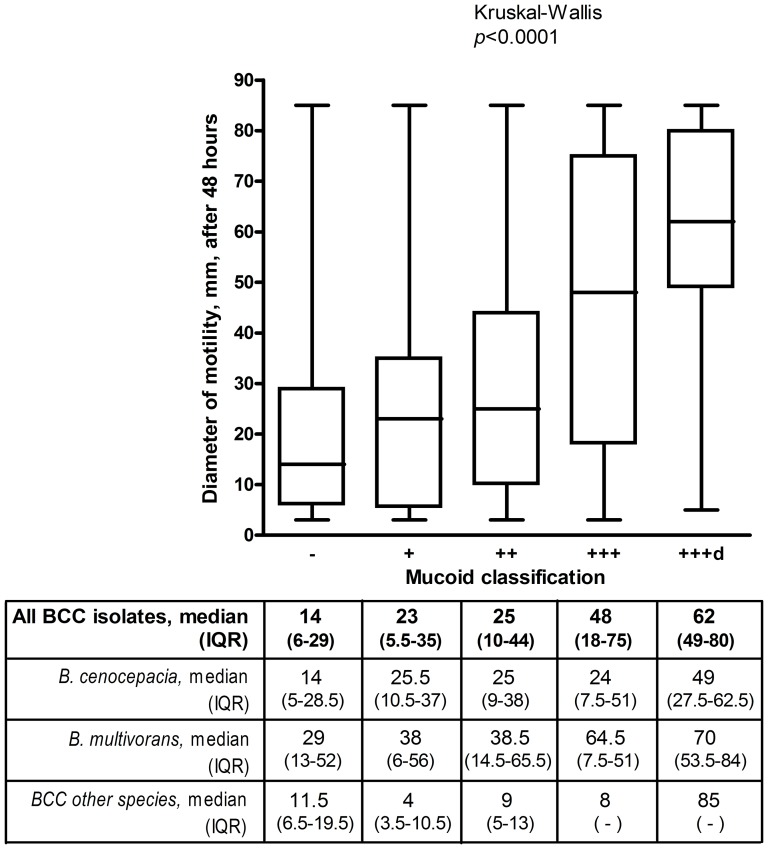
Motility and the mucoid phenotype in BCC. Box and Whisker plot of motility phenotype compared to the mucoid phenotype (as previously assessed: [23]) in all isolates of *B. cepacia* complex assessed for which we had corresponding mucoid scores (*n = *541). Statistical significance was assessed by the non parametic Kruskal-Wallis one-way analysis of variance in GraphPad Prism v.4. Data were also analyzed separately for *B. cenocepacia* (*n* = 288) and *B. multivorans* (*n* = 203) as well as all the remaining species examined (*n* = 50) and the medians and inter-quartile range (IQR) are provided in the box below the figure. IQRs could not be calculated for BCC other species scored +++ and +++d because there were only 3 and 1 isolates in those categories respectively.

Previous studies of clonal variants in *B. cenocepacia* taken from a CF lung infection have found that nonmucoid variants demonstrated loss of swimming motility capacity [24], [38]. Unlike *P. aeruginosa*, the mucoid phenotype is not visually detectable on LB-agar motility plates [23]. Therefore any differences observed in the diameter of growth on swimming motility plates used in this study is due to the swimming motility phenotype, rather than excess elaboration of exopolysaccharide in mucoid isolates. Even though the mucoid phenotype does not manifest itself visually on LB agar plates it is conceivable there still could be a link between EPS production capacity and swimming motility. As we have previously assessed the mucoid phenotype in these isolates [23], we examined the swimming motility phenotype in relation to the mucoid phenotype. These data support previous observations, showing a general, but not absolute, correlation between the extent of mucoidy, scored from – to +++ and capacity for swimming motility as measured by the diameter of bacterial growth after 48 hours at 35°C (Fig 2). This trend was statistically significant (Kruskal-Wallis *p*<0.0001). Breaking down these data into species, the same trend was observable for both *B. multivorans* and *B. cenocepacia* (Fig 2), which was statistically significant for both species (data not shown).

### The motility phenotype in relation to clinical outcome in BCC lung infections in people with cystic fibrosis

**Figure 3:**
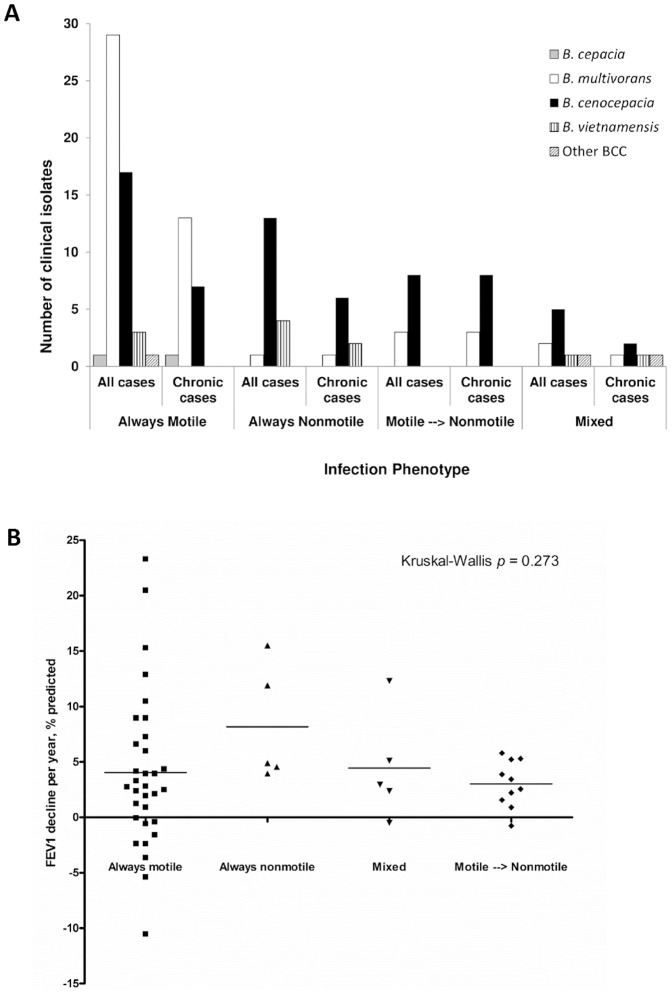
Assessment of clinical significance of swimming motility in BCC bacteria. A) CF lung infections categorized by species and motility phenotype. Data are displayed for all cases (*n* = 89) and for cases where chronic infection (*n* = 48) was clearly established (defined as the presence in our collection of at least 2 or more isolates of the same strain type that were collected at least a year apart). Grey bars  =  *B. cepacia*, white bars  =  *B. multivorans*, black bars  =  *B. cenocepacia*; vertical striped bars  =  *B. vietnamiensis* and diagonal striped bars  =  other BCC species. Infections were categorized as: isolates were always motile; always nonmotile; motile to nonmotile phenotype switches detected during infection (as defined by an initial isolation of at least 2 separate motile isolates on separate months followed by a nonmotile isolate at any point thereafter) or mixed (there was no clear established initial swimming motility phenotype as the first two isolates stored for patients on separate months demonstrated both motile and nonmotile phenotypes). One case was also classified as mixed (FEV1% predicted decline −0.5%) as most isolates were nonmotile, however several marginally motile (diameter 11 and 12 mm) isolates were detected during the course of the patients' infection. B) Assessment of the impact of motility phenotype on FEV1 decline following initial infection with BCC. FEV1 data were collected from the patient charts as described previously. Criteria for inclusion were established previously [22]. Data were analyzed in GraphPad Prism v.4 and the non-parametric Kruskal-Wallis test calculated to assess difference.

Given that initial colonizing isolates of *P. aeruginosa* infection in CF are typically motile, while phenotype switches to nonmotility are commonly seen in later isolates, we were interested in assessing the clinical significance of the BCC swimming motility phenotype. In the context of the 89 clinical infections where there was infection by a single clone, 51/89 infections only ever had motile BCC isolated while 19/89 were only ever nonmotile, indicating that nonmotile isolates of BCC may be competent to initiate and maintain infection (Fig. 3A). We also examined the dataset to look at infections where there was clear evidence of chronic infection (defined as the presence in our collection of at least three different isolates of the same strain type over the period of at least a year, *n* = 48). Swimming motility phenotype switches were detected during chronic infection, but only in a minority of cases (11/48). Like *P. aeruginosa*, these switches were always motile to nonmotile, although they were often accompanied by subsequent isolation of motile variants (data not shown). Previous RAPD typing of these isolates [23] indicated that all cases of swimming motility phenotype switching took place in sequential clonal isolates and therefore were not replacement infections (data not shown). In total these data represent over 214 patient years of BCC infection data and an average of 8.5 isolates per patient for chronic infections.

We also sought to assess the clinical impact of the different phenotypes (Fig 3B). Patient data were previously collected and this study was approved by the UBC research ethics boards [22]. For cases for which we have suitable follow up clinical data (*n* = 52), as defined by our previous criteria [22], we assessed the post infection decline in lung function, using Forced Expiratory Volume in one second (FEV1), % predicted. The mean annual post-infection lung function decline was 4.0% FEV1 % predicted for those infected with motile BCC bacteria (*n* = 32), while those only infected with nonmotile BCC bacteria (*n* = 5) had a mean annual decline of 8.2% FEV1% predicted. Patients who were colonized with either mixed motile phenotypes (*n* = 5) or those where there was clear evidence of a motile to nonmotile phenotype switch (*n* = 10) had annual post-infection FEV1% predicted declines of 4.4% and 3.0% respectively. Among all groups, these data demonstrated no significant differences between patients infected with motile or nonmotile BCC, irrespective of whether we examined all cases (Kruskal-Wallis *p* = 0.273, Fig. 3B) or just those for whom chronic infection (defined as the presence in our collection of 3 or more isolates over a period of a year or more) was documented (*n* = 34, Kruskal-Wallis *p* = 0.174, data not shown).

### The effects of loss of motility on the biological properties of BCC bacteria during chronic infection

To assess the biological consequence of the motile to nonmotile switches, we investigated the production of flagellum protein by Western blot using a polyclonal antibody [36] raised against *B. pseudomallei* flagellin, as described previously [37]. We examined isolates from 5 patients in whom there was a switch from motility to nonmotility in later isolates. These cases were selected to represent *B. multivorans* as well as most of the major epidemic lineages of *B. cenocepacia* (RAPD types 01, 04 and 06). In addition, we also examined a well-described isolate of the RAPD02/ET-12 lineage, K56-2. In most cases, lack of motility was associated with a loss of detectable flagellin protein, however flagellin protein was detected in 2 nonmotile isolates (C9343 and VC8402) from two patients in which there had been a phenotype switch from motile to nonmotile (Table 1). Given the role flagella play in activating inflammation through the pattern recognition receptor TLR5, we also assessed TLR5 activation in these isolates using a TLR5-NF-κB luciferase reporter assay system as described previously [32]. Motile isolates demonstrated significantly increased TLR5 activation compared to nonmotile isolates (2.88 vs 2.10 fold compared to unstimulated controls, unpaired one tailed *t*-test *p* = 0.0092).

## Discussion

Chronic bacterial infections in CF are particularly difficult to model *ex-vivo*, in part due to the length of time that bacteria are resident in the CF lung, making it hard to mimic conditions of the CF lung using either *in vitro* or animal/plant model systems. The inability to effectively model CF lung infections in the laboratory makes it challenging to understand the contribution any given bacterial trait may have towards disease severity or to understand the adaptive mechanisms bacteria use to facilitate chronic residence in the CF lung. There are a large range of putative virulence determinants now described for BCC bacteria, for a recent review see [39], however, for the most part, the clinical significance of these determinants is unclear. In addition, there is a growing range of *ex-vivo* models for BCC infection, however there is little consistency among these models in terms of conserved virulence determinants and in any case none of the models truly mimics CF infection [40].

Swimming motility is well described as an adaptive trait in *P. aeruginosa* infections in CF whereby initial infection is typically by a motile isolate which can convert to a nonmotile phenotype later in chronic infection [11], [14]. The swimming motility phenotype is also well described in BCC bacteria and is mediated by a single polar flagellum [41]–[43]. *In-vitro* experiments have shown that the BCC flagellum facilitates invasion into epithelial cells *in-vitro* and promotion of inflammatory markers [34], [44]. The clinical significance of motility in chronic BCC lung infections in CF, however, is not clear.

In order to describe the significance of motility in clinical isolates of BCC we examined our collection of BCC taken on a regular basis from people with CF. Aside from the longitudinal nature of our BCC collection, a strength of this study is that our collection contains large numbers of the two most commonly isolated species from the BCC: *B. cenocepacia* and *B. multivorans*. Our data reveal that both motile and nonmotile isolates can be recovered from CF lung infections for all species examined, except for *B. cepacia* for which we only had 4 isolates from a single infection and *B. metallica* for which we had only one isolate (Table 1). Stratifying infections into ‘motility phenotypes’ revealed that isolates from the majority of chronic infections most commonly display the motile phenotype throughout the infection and do not normally switch to nonmotile (Fig 3A). We identified a limited number of infections where nonmotile isolates were the only phenotype ever isolated, suggesting that nonmotile BCC bacteria are also competent to initiate infection. However, these data must be understood in the context that although they were the first recovered isolates, the duration of colonization prior to the first positive culture cannot be determined. Additionally, although there was minimal passage of the bacteria between the lung and storage, it is still conceivable that phenotype switching may have already taken place as a consequence of adaptation to growth either in the lung or in the lab. Nonetheless, by examining archived bacterial isolates, the approach we have taken in this study is identical to that used by us to describe the evolution of *P. aeruginosa* motility in the CF lung [11].

A number of studies have examined adaptation of BCC bacteria to various *in-vitro* growth environments [45]–[49]. However, there are currently little data on adaptive strategies of BCC bacteria to chronic pulmonary infection in CF. This study adds to other evidence [16], [22] which suggest that at least some of the mechanisms of pathogenesis in BCC bacterial lung disease are distinct from those described for *P. aeruginosa*. Our data indicate that loss of motility is not a common adaptive feature in BCC bacteria to chronic lung infection, as opposed to *P. aeruginosa* (Fig 3A). Examination of the biological properties of sequential clonal isolates from infections where there was switch from motile to nonmotile phenotypes (Table 2), revealed that loss of motility is often, but not always, associated with a loss of detectable flagellin protein by Western blot. Consistent with published data [33], we also show that conversion to the nonmotile phenotype was associated with a reduction in TLR-5 stimulator capacity (Table 2).

**Table 2:** Biological characteristics of motile and nonmotile CF clinical isolates of *Burkholderia cepacia* complex bacteria.

Patient	Isolate	Species	Date of isolation	Strain type (RAPD)	Motility	Flagella Western blot	TLR5 activity
A	C3921	BC	Oct 1990	001	+	+	1.95 (0.44)
	C8963	BC	Jan 2000	001	−	−	2.00 (0.27)
	C9343	BC	Oct 2000	001	−	+	2.99 (0.56)
B	VC3868	BC	Aug 1990	004	+	+	3.35 (0.69)
	VC9670	BC	Jun 1999	004	−	−	1.82 (0.26)
	VC13450	BC	Jul 2006	004	−	−	1.99 (0.29)
C	VC6432	BC	Jul 1994	006	+	+	3.38 (0.78)
	VC8402	BC	Sep 1997	006	−	+	2.47 (0.59)
D	VC2307	BC	Apr 1987	006	+	+	3.71 (1.00)
	VC6905	BC	Mar 1995	006	−	−	1.82 (1.00)
E	VC12201	BC	Jan 2004	009	+	+	3.10 (0.58)
	VC13307	BC	Mar 2006	009	−	−	1.87 (0.25)
F	VC12539	BM	Sep 2004	BM-044	+	+	1.86 (0.25)
	VC13732	BM	Mar 2007	BM-044	−	−	1.85 (0.32)
n/a	K56-2	BC	−	002	+	+	3.03 (0.41)

Date of isolation  =  month/year. Species: BC  =  *B. cenocepacia*, BM  =  *B. multivorans*. Strain types were determined previously for these isolates by RAPD [23], [50]. Western blotting was performed on whole cell extracts using a polyclonal anti-*B. pseudomallei* flagella antibody [36] as we have described previously [37]. Results are representative of three independent experiments and are positive or negative for the presence of a band at either 45 kDa or 55 kDa – the variants of flagella known to be produced by BCC bacteria [41]. TLR5 activation values are the mean of assays performed in triplicate on five separate occasions, with standard error of the mean in brackets.

These data further support the contention that the adaptive strategies used by BCC in chronic CF lung infections are distinct from those described for *P. aeruginosa* and therefore BCC merits separate consideration. While phenotype conversion, from motile to nonmotile, did occur during chronic infection, it was not common and was often accompanied by the subsequent isolation of motile isolates from the same infection (Figure 3A). Other data from this isolate collection also supports this suggestion. One study has shown that BCC maintain functional quorum sensing signaling systems during chronic infection, whereas isolation of quorum sensing mutants during chronic *P. aeruginosa* infections in CF is common [16], [22]. Meanwhile we have also shown that conversion to the nonmucoid phenotype in BCC infections is common, while the extent of mucoidy is inversely related to survival and outcome, also in contrast to *P. aeruginosa* [22], [23].

Our data are supportive of a link between motility and the mucoid phenotype as has previously been described by others [24], [38]. This correlation was seen both when all isolates were examined and in both *B. multivorans* and *B. cenocepacia* isolates when examined separately (Figure 2). Nonetheless, it is notable that there is considerable variation in all mucoid classes in their capacity for swimming motility and we identified both nonmotile and motile nonmucoid isolates (considered as mucoid scores – and +) as well as nonmotile and motile mucoid isolates (considered as mucoid scores ++/+++/+++d) (Fig 2). Therefore our data indicate that, while there is a significant trend, the connection between swimming motility and the mucoid colonial morphology phenotype is not absolute. Given our previously described link between mucoidy and outcome [22], [23], the link described here between mucoidy and motility might suggest that there would be a link with patient outcome in terms of lung function decline. However, we did not observe a link between motility and clinical outcome (Fig 3B). Conclusions from these data are limited by the small numbers of cases in the nonmotile group in particular (*n* = 5) and therefore this question requires further investigation in another population containing larger numbers of nonmotile infections.

We believe this study is the most comprehensive analysis to date of the swimming motility phenotype in clinical isolates of BCC bacteria. Based on these data, it appears that differences in the motility phenotype do not contribute significantly to the severity of BCC lung disease in CF. We suggest, however, that these data and others indicate that the pathogenic mechanisms of BCC bacteria in CF lung disease need to be considered separately from those that have been observed for *P. aeruginosa*. The assertion is supported by the clinical picture, where BCC have a well-established elevated risk of mortality/lung transplantation and can result in an invasive necrotizing pneumonia known as ‘cepacia syndrome’ rarely if ever seen in *P. aeruginosa* infections [3], [20]. As a consequence further studies on adaptation to the CF lung, using clinical isolates with matched clinical data from patients are merited.
